# Lanthanum-Modified Sludge Biochar for Geothermal Water Fluoride Removal

**DOI:** 10.3390/ma18071421

**Published:** 2025-03-23

**Authors:** Wei Li, Yi Wu, Ruiqing Huang, Chen Yang, Mei Zhang, Pengchen Xie, Jian Xiong, Xuebin Lu

**Affiliations:** 1 School of Ecology and Environment, Tibet University, Lhasa 850012, China; weili_xzdx@163.com (W.L.); yiw_0000@126.com (Y.W.); 17320504779@163.com (R.H.); yangchen02468@126.com (C.Y.); 15826471431@163.com (M.Z.); xpc15121632897@163.com (P.X.); jianxiong@utibet.edu.cn (J.X.); 2 School of Environmental Science and Engineering, Tianjin University, Tianjin 300350, China

**Keywords:** sludge biochar, geothermal water, defluorination, adsorption

## Abstract

Municipal sludge was pyrolyzed to produce sludge-derived biochar (SBC), which was subsequently modified with lanthanum nitrate. Through orthogonal experiments, the optimal preparation conditions for La-SBC-700 were determined. The morphological and textural properties of the biochar material, such as specific surface area, were characterized and batch adsorption experiments simulating fluoride-containing wastewater were conducted to investigate the effects of pH, fluoride concentration, biochar dosage, and coexisting ions on the fluoride removal performance of La-SBC-700. The application potential of the biochar material in real geothermal water was also assessed. The results indicated that La-SBC-700 prepared under optimal conditions exhibited an adsorption capacity approximately 10 times higher than that of the pristine biochar (SBC). The adsorption process was stable within the pH range of 5.0 to 8.0 and conformed to the Quasi-secondary-order kinetic and Langmuir isotherm models, with a maximum theoretical adsorption capacity of 40.338 mg/g. The adsorption process was spontaneous and endothermic. ${NO}_{3}^{-}$ and Cl^−^ had negligible effects on fluoride removal, whereas ${CO}_{3}^{2-}$, ${SO}_{4}^{2-}$, and ${HCO}_{3}^{-}$ exerted varying degrees of influence on the adsorption process. La-SBC-700 demonstrated excellent performance in removing fluoride from geothermal water, providing a reference method for the resourceful utilization of sludge and the removal of fluoride from geothermal water.

## 1. Introduction

Fluorine (F) is widely present in nature and is one of the essential trace elements for the human body. However, the excessive intake of fluorine can cause many harmful effects on human health. Approximately 23 countries and 200 million people worldwide are threatened by fluorine pollution [1]. Geothermal resources are a new type of energy with great potential for development. Studies have shown that the concentration of fluorides in geothermal fields and groundwater is closely related to them. For example, in Yangbajing Town, Tibet, China, where geothermal hot spring resources are relatively concentrated, the fluoride concentration in geothermal well water reaches 19.4 mg/L [2]. The detection rate of fluorides in some surrounding water areas and downstream river sections is also relatively high [3]. As one of the important indicators in water quality monitoring, the World Health Organization (WHO) stipulates that the maximum concentration of F^−^ in drinking water is 1.5 mg·L^−1^ [4]. Therefore, exploring methods for removing fluorine from geothermal water is of great significance for both water ecological environment and human health.

Current methods for fluoride removal from aqueous solutions can be categorized into precipitation–coagulation techniques, membrane separation processes, ion exchange methods, and adsorption approaches [5,6,7,8]. Among these, adsorption has garnered considerable attention due to its cost-effectiveness and high removal efficiency. Biochar, characterized by its abundant surface functional groups, stable structure, and wide availability of raw materials, has emerged as a sustainable and promising adsorbent for aquatic environmental remediation. The biochar prepared from Indian wood orange peel by Kalpana Singh [9] and applied to fluoride-containing wastewater has a maximum adsorption capacity of 2.40 mg/g.

Mohanta [10] demonstrated that SnO_2_-loaded biochar derived from phosphoric acid-activated carbonized sawdust achieved a maximum fluoride adsorption capacity of 4.60 mg/g. In contrast, Yu [11] reported a superior adsorption performance using lanthanum-modified biochar synthesized through the concentrated sulfuric acid pretreatment of Sargassum biomass, which exhibited an exceptional fluoride adsorption capacity of 94.344 mg/g under neutral pH conditions. The fluoride adsorption capacities of biochar exhibit significant variation depending on raw material sources and preparation methodologies. Due to their inherent negatively charged surfaces, biochar materials generally demonstrate weak electrostatic interactions with anionic contaminants like F^−^, resulting in limited adsorption efficiency. To address this limitation, the strategic incorporation of metal ions into pristine biochar matrices has been widely adopted to enhance adsorption performance toward anions, as evidenced in recent studies [12].

Studies have shown that lanthanum (La) has a high affinity for F^−^ [13]. For instance, VENCES-ALVAREZ et al. [14] modified commercial activated carbon with La^3+^ solution, achieving a maximum fluoride adsorption capacity of 9.96 mg/g. Wang [15] et al. mixed pomelo peel biochar (PPBC) with La(NO_3_)_3_ solution to produce a lanthanum-modified biochar (La-PPBC), which showed a maximum fluoride adsorption capacity of 19.86 mg/g. Zhou et al. [16] loaded La/Fe/Al oxides onto rice straw biochar (RSBC) to create a new adsorbent (La/Fe/Al-RSBC) with an adsorption capacity up to 111.11 mg/g. These methods successfully loaded lanthanum (La) onto biochar, increasing the surface functional groups and enhancing the affinity for fluoride [17]. However, they also face challenges such as complex preparation processes, high costs, and low loading efficiencies.

Municipal sludge (referred to as sludge) has a complex composition and is toxic. Current treatment methods mainly include sanitary landfill, incineration, and composting, but these methods carry the risk of secondary pollution [18], increasing waste treatment costs and causing resource waste. Sludge contains a large amount of minerals that can provide more adsorption sites, but due to the original carbon structure, the adsorption rate for pollutants is low. Therefore, FENG et al. [19] loaded metals onto sludge biochar, achieving a maximum adsorption capacity of up to 30.49 mg/g.

This research and development was rooted in the investigation of lanthanum-modified fluoride removal materials derived from sludge biochar, with the aim of expanding the resource utilization pathways within sludge systems. Through orthogonal experiments, the optimal preparation conditions for lanthanum-loaded sludge biochar were determined. The changes in sludge-based biochar before and after modification were analyzed, and the adsorption efficiency of lanthanum-loaded sludge biochar for fluoride under different experimental conditions was simulated, aiming to provide basic data support for fluoride removal technology in geothermal water.

## 2. Materials and Methods

### 2.1. Materials, Reagents, and Instruments

The sludge used in this study was collected from a wastewater treatment plant in Lhasa, Tibet, China. The sludge was air-dried naturally, then dried in an oven at 105 °C for 24 h, crushed, and sieved through a 100-mesh sieve. It was further dried at 105 °C for another 24 h, sealed in sample bags, and stored for later use.

Reagents: (La(NO_3_)_3_·6H_2_O), NaF, NaOH, HCI, Na_2_CO_3_, NaHCO_3_, NaCl, Na_2_SO_4_, NaNO_3_. All of the analytical-grade reagents were purchased from Aladdin Reagent Co., Ltd. (Aladdin Biochemical Technology Co., Ltd., Shanghai, China). Deionized water was used throughout the experiments.

Instruments: Fluoride ion meter (PXSJ-216F, Accuracy of 0.001pX, Shanghai Yidian Scientific Instrument Co., Ltd., Shanghai, China); tube furnace (TFH-1200-50-200, Accuracy of 0.1 °C, Anhui Kemi Instrument Co., Ltd., Hefei, China).

### 2.2. Experimental Methods

#### 2.2.1. Determination of Fluoride Concentration

The concentration of fluoride in water was determined using the electrode method. A standard curve was plotted based on the Nernst equation, where the fluoride potential value E (in mV) is directly proportional to the negative logarithm of the fluoride concentration(−lgCe), and a standard curve was plotted. In this study, the standard curve equation for fluoride was E = k(−lgCe) + b (specifically, y = 58.77x + 251.77), with a correlation coefficient of R^2^ = 0.99996. When measuring the fluoride concentration, 3 mL of an ionic strength adjuster was added to the sample solution. The fluoride concentration of the sample could then be directly measured at a constant temperature of 25 °C.

#### 2.2.2. Production of Biochar

The pretreated sludge was placed into a tubular furnace, and then nitrogen gas (N_2_) was introduced. The heating rate was set at 7 °C/min, and the pyrolysis time was 1 h. The pyrolysis temperatures were set at 300, 500, 700, and 750 °C, respectively. Sludge-based biochar prepared at these different temperatures was labeled as SBC-300, SBC-500, SBC-700, and SBC-750.

Based on the preparation of pristine biochar, the effect of different factor levels (modification temperatures were set at 35 °C, 45 °C, and 55 °C; pH values of lanthanum nitrate solution were set at 7, 9, and 11; the solid–liquid ratio of biochar to lanthanum nitrate solution was set at 1:15, 1:20, and 1:25; and the concentration of lanthanum nitrate was set at 0.04 mol/L, 0.06 mol/L, and 0.08 mol/L) on the resulting lanthanum-loaded sludge biochar was investigated, denoted as La-SBC.

An orthogonal experiment was designed with fluoride adsorption capacity as the evaluation index. The main and secondary relationships of different influencing factors were analyzed using the range analysis method to determine the optimal experimental combination for the preparation of lanthanum-loaded biochar.

#### 2.2.3. Characterization of Biochar Samples

The samples were attached to conductive adhesive and then sputter-coated with gold. The morphologies of the sludge biochar and the elements on the surface were observed using a field emission scanning electron microscope (SEM-EDS) (SU 8020, Hitachi, Chiyoda City, Japan). The experimental parameters were as follows: voltage 2.5 kV to 3.5 kV; magnification size 1 μm.

Fourier-Transform Infrared Spectroscopy (FTIR) (Great 20, Zhongke Ruijie Technology Co., Ltd., Tianjin, China) was used to analyze the changes in the functional group structure on the surface of the biochar. The experimental parameters were scanning range of 500 to 4000 cm^−1^, resolution of 4 cm^−1^, and 32 scans.

A specific surface area and pore size analyzer (BET) (V-sorb 2800TP, with a repeatability precision of specific surface area ≤ ±1.0%, pore size repeatability deviation ≤ 0.02 nm, Beijing Guoyi Precision Measurement Technology Co., Ltd., Beijing, China) was used to analyze the specific surface area, pore volume, and pore size of the biochar. The experimental parameters were 200 °C/4 h (pretreatment temperature and time); −196 °C (liquid nitrogen environment).

An X-ray diffractometer (XRD) (TD-3700, Dandong Tongda Technology Co., Ltd., Dandong, China) was used to analyze the types of phases present in the biochar samples. The experimental parameters were angular momentum increment of 0.08°; sampling time of 1 s.

#### 2.2.4. Design of Batch Adsorption Experiments

##### Adsorption Experiments with Sludge-Derived Biochar

Several centrifuge tubes were taken, and 19 mg/L simulated fluoride wastewater (prepared from sodium fluoride) with a pH of 7 was added to each of them. Then, 0.1000 g of SBC-300, SBC-500, SBC-700, and SBC-750 biochars was added, respectively. The tubes were agitated at 180 r/min for 8 h using a constant temperature shaker. The adsorption experiment was carried out at 40 °C. The supernatant was collected, filtered through a 0.45 μm membrane, and the concentration of fluoride in the filtrate was measured using a fluoride ion-selective electrode (PXSJ-216F). Finally, the removal efficiency of fluoride and the adsorption capacity were calculated using Equations (1) and (2), respectively. The experiment was conducted with three parallel tests.

##### Adsorption Experiments with Lanthanum-Loaded Sludge Biochar

Several centrifuge tubes were taken, and 40 mL of 19 mg/L simulated fluoride wastewater was added to each. Lanthanum-loaded sludge biochar weighing 0.1000 g was added, and the pH of the solution was adjusted to 5, 6, 7, 8, 9, 10, 11, and 12, respectively. The tubes were agitated at 180 r/min for 8 h using a constant temperature shaker at 40 °C for the adsorption experiment. The supernatant was collected, filtered through a 0.45 μm membrane, and the concentration of fluoride in the filtrate was measured using a fluoride ion-selective electrode (PXSJ-216F). The removal efficiency of fluoride and the adsorption capacity were calculated separately to determine the optimal pH.

Several centrifuge tubes were taken, and 40 mL of 19 mg/L simulated fluoride wastewater was added to each. A total of 0.1000 g of lanthanum-loaded sludge biochar was added, and the pH of the solution was adjusted to the optimal level. The tubes were agitated at 180 r/min for 8 h using a constant temperature shaker, and adsorption experiments were conducted at 20, 40, 60, and 80 °C, respectively. The supernatant was collected, filtered through a 0.45 μm membrane, and the concentration of fluoride in the filtrate was measured using a fluoride ion-selective electrode (PXSJ-216F). The removal efficiency of fluoride and the adsorption capacity were calculated separately to determine the optimal temperature.

Several centrifuge tubes were taken, and 40 mL of 19 mg/L simulated fluoride wastewater at the optimal pH was added to each. Appropriate amounts of lanthanum-loaded sludge biochar (0.0100, 0.0200, 0.0300, 0.0400, 0.0500, 0.0600, 0.0700, 0.0800, 0.0900, 0.1000 g) were added, respectively. The tubes were agitated at 180 r/min for 8 h using a constant temperature shaker at the optimal temperature for the adsorption experiment. The supernatant was collected, filtered through a 0.45 μm membrane, and the concentration of fluoride in the filtrate was measured using a fluoride ion-selective electrode (PXSJ-216F). The removal efficiency of fluoride and the adsorption capacity were calculated separately to determine the optimal amount of biochar to be added.

Kinetic adsorption experimental conditions: Several centrifuge tubes were taken, and 40 mL of 19 mg/L simulated fluoride wastewater, with the pH adjusted to the optimal level, was added to each. The optimal amount of lanthanum-loaded sludge biochar was added, and the tubes were agitated at 180 r/min using a constant temperature shaker. Different times (0.17, 0.33, 0.5, 1, 2, 5, 10, 30, 60, 120, 180, 240, 300, 360, 420, 480 min) were set for the adsorption experiment at the optimal temperature. The supernatant was collected, filtered through a 0.45 μm membrane, and the concentration of fluoride in the filtrate was measured using a fluoride ion-selective electrode (PXSJ-216F). The removal efficiency of fluoride and the adsorption capacity were calculated separately.

Isothermal adsorption and thermodynamic experimental conditions: Several centrifuge tubes were taken, and 40 mL of simulated fluoride wastewater at different concentrations (20, 40, 60, 80, 100, 120 mg/L) was added to each. The pH was adjusted to the optimal level, and the optimal amount of lanthanum-loaded sludge biochar was added. The tubes were agitated at 180 r/min for 24 h using a constant temperature shaker at different temperatures (20, 40, 60, 80 °C). The supernatant was collected, filtered through a 0.45 μm membrane, and the concentration of fluoride in the filtrate was measured using a fluoride ion-selective electrode (PXSJ-216F). The removal efficiency of fluoride and the adsorption capacity were calculated separately.

All the above experiments were conducted with three replicates.

#### 2.2.5. Coexisting Anion Interference Experiments

Considering that the pH of geothermal water is generally neutral to slightly alkaline, the effects of five common anions (Cl^−^, ${NO}_{3}^{-}$, ${SO}_{4}^{2-}$, ${HCO}_{3}^{-}$, and ${HCO}_{3}^{2-}$) on the adsorption efficiency of lanthanum-loaded sludge biochar (La-SBC) were evaluated. The solutions were binary systems containing fluoride and one of the anions. Appropriate amounts of coexisting anions (50, 100, and 200 mg/L) were combined with a suitable amount of La-SBC. The mixtures were oscillated at a water bath temperature of 40 °C for 8 h. The supernatant was then taken and filtered through a 0.45 μm filter membrane. The potential value was measured, and the fluoride concentration was calculated based on the potential fluoride ion concentration standard curve. The removal rate and adsorption capacity of fluoride were also calculated.

#### 2.2.6. Data Processing

The removal rate of fluoride (Equation (1)) and the amount adsorbed at adsorption equilibrium (Equation (2)):

(1) $$\eta =\frac{\left(C_{0}-C_{e}\right)}{C_{0}}\times 100\%$$

(2) $$q=\frac{\left(C_{0}-C_{e}\right)\times V}{m}$$

In the equations, *η* represents the removal efficiency of fluoride (%); *q* represents the adsorption capacity of the adsorbent for fluoride (mg/g); *C*_0_ represents the initial concentration of fluoride (mg/L); *C_e_* represents the fluoride concentration at adsorption equilibrium (mg/L); *m* represents the dosage of SBC (La-SBC) (g); and *V* represents the volume of the fluoride solution (mL).

The adsorption kinetic data were fitted using the Quasi-primary kinetics equation (Equation (3)) and the Quasi-secondary kinetics equations (Equation (4)).

The Quasi-primary-order kinetics equation is as follows:

(3) $$q_{t}=q_{e}\left(1-e^{-k_{1}t}\right)$$

The Quasi-second-order kinetics equation is as follows:

(4) $$q_{t}=\frac{q_{e}^{2}k_{2}t}{1+q_{e}k_{2}t}$$

In the equations, *q_t_* and *q_e_* represent the fluoride adsorption capacities at time t and at theoretical equilibrium, respectively (mg/g); *t* represents the adsorption time (min); *k*_1_ represents the rate constant of the Quasi-primary-order kinetics constant (min^−1^); and *k*_2_ represents the rate constant of the Quasi-secondary-order kinetic constant in g·(mg·min)^−1^.

The isothermal adsorption data were fitted using the Langmuir and Freundlich isotherm models, represented by Equations (5) and (6), respectively,

(5) $$q_{e}=\frac{q_{m}K_{L}C_{e}}{1+K_{L}C_{e}}$$

(6) $$q_{e}=K_{F}C_{e}^{1/n}$$

In the equations, *q_m_* represents the maximum monolayer adsorption capacity (mg/g); *q_e_* represents the fluoride adsorption capacity at equilibrium (mg/g); *C_e_* represents the fluoride concentration at equilibrium (mg/L); *K_L_* represents the Langmuir adsorption equilibrium constant (L/mg); *K_F_* represents the Freundlich adsorption equilibrium constant (mg·L ^1/n^). (g·mg^1/n^)^−1^; and *n* represents the adsorption intensity index.

The standard Gibbs free energy change(Δ*G*^0^), standard enthalpy change (Δ*H*^0^), and standard entropy change (Δ*S*^0^) were calculated using Equations (7), (8), and (9), respectively.

(7) $$K_{0}=\frac{q_{e}}{C_{e}}$$

(8) $$\Delta G^{0}=-RT\ln K_{0}$$

(9) $$\ln K_{0}=\frac{\Delta S^{0}}{R}-\frac{\Delta H^{0}}{RT}$$

Herein, *R* represents the ideal gas constant, 8.314 kJ/(mol·K); *T* represents the absolute temperature (K); *K*_0_ represents the thermodynamic equilibrium constant; *q_e_* represents the fluoride adsorption capacity at equilibrium, in mg/g; *C_e_* represents the fluoride concentration at adsorption equilibrium (mg/L); Δ*G*^0^ represents the standard Gibbs free energy change, in kJ/mol; Δ*H*^0^ represents the standard enthalpy change, in kJ/mol; and Δ*S*^0^ represents the standard entropy change, in kJ/(mol·K).

In this study, all data calculations were performed using Excel 2019, while data visualization, graphing, model fitting, and statistical analysis were conducted using Origin 2021pro.

## 3. Results and Discussion

### 3.1. Preparation and Characterization of Sludge Biochar

#### 3.1.1. Preparation of Biochar

Using sludge as the raw material, biochar was produced through pyrolysis at specific temperatures, and its adsorption capacity for fluoride was explored. It was found that the adsorption capacity of biochar for fluoride increased with the rise in pyrolysis temperature. When the temperature reached 700 °C, the adsorption rate became stable. The maximum adsorption capacity of SBC-700 for fluoride reached 0.64 mg/g. This is because sludge contains a large amount of organic matter. As the pyrolysis temperature increases, the decomposition of organic matter produces gases, which gradually enrich the pore structure of the biochar and increase its specific surface area, thereby enhancing the adsorption capacity of the material [20].

#### 3.1.2. Characterization of Biochar

##### Scanning Electron Microscopy and X-Ray Photoelectron Spectroscopy Analysis

**Figure 1:**
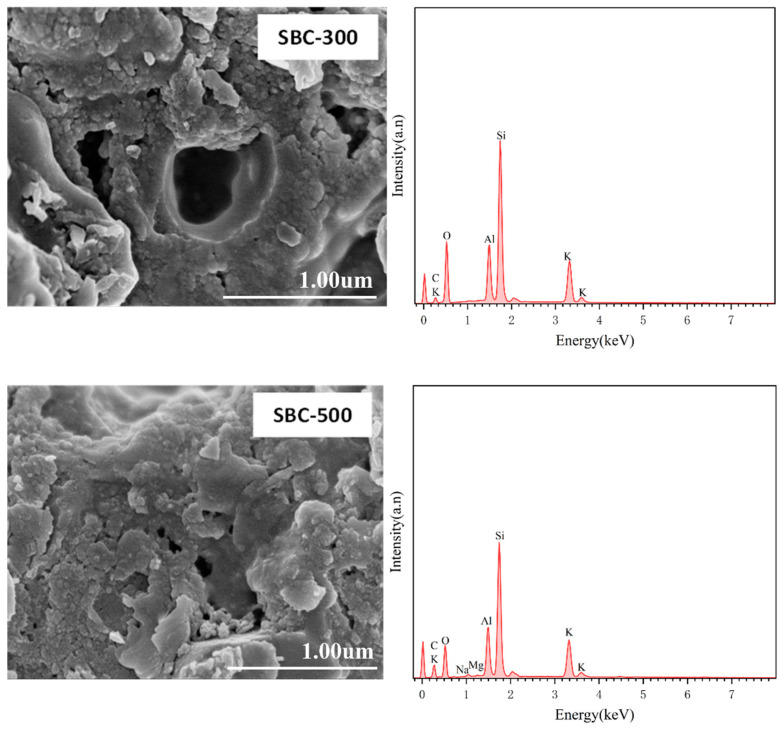

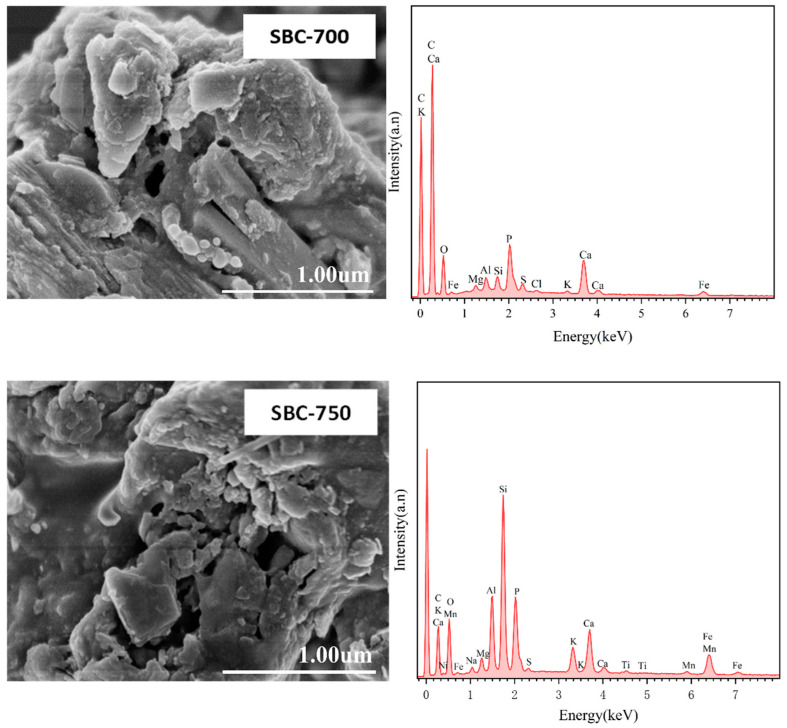
SEM-EDS images of sludge biochar (SBC) prepared at different temperatures.

As shown in Figure 1, as the pyrolysis temperature increased, the porosity of the biochar surface also increased. The EDS images revealed that the surface elements of the biochar were complex. For the SBC-300 and SBC-500 samples, the main surface elements are Si, O, C, and Al, with trace amounts of K. When the pyrolysis temperature increased to 700 °C and above, new elements such as Ca, Mn, and Fe appeared in the SBC-700 and SBC-750 samples, indicating that as the pyrolysis temperature increased, organic matter in the sludge volatilized, while various metal elements attached to the sludge were retained.

##### Comparative Surface Area and Aperture Analysis

Specific surface area and pore size are crucial parameters affecting the adsorption performance of biochar. The specific surface area, pore size, and other parameters of the pristine sludge biochar were measured, and the results are shown in Table 1.

As shown in Table 1, the specific surface area, micropore area, total pore volume, and micropore volume of the pristine biochar initially increased and then decreased as the pyrolysis temperature increased. Among them, SBC-700 has a particularly high specific surface area of 39.632 m^2^/g, indicating a rich pore structure dominated by micropores. This is because the sludge contains a large amount of water, which forms steam during pyrolysis, and the decomposition of organic matter, cellulose, and other organic compounds generates a significant amount of gas, creating a high proportion of micropores within the biochar [21]. As the pyrolysis temperature increases, more new micropores are formed [22]. However, when the temperature exceeds 700 °C, parameters such as the specific surface area and total pore volume of SBC-750 show a certain downward trend, while the average pore diameter increases. This is due to the collapse of the biochar’s pore structure framework at excessively high temperatures, which leads to a decrease in specific surface area and an increase in average pore diameter and total pore volume [23].

##### Infrared Spectroscopic Analysis

**Figure 2:**
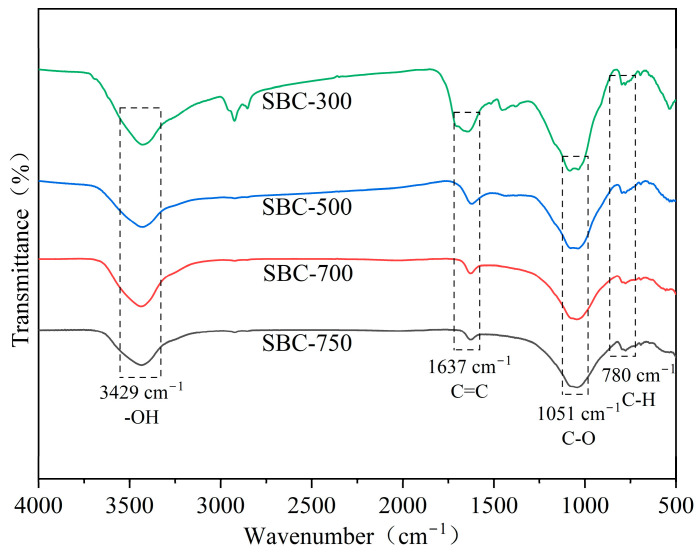
FTIR image of sludge biochar.

The FTIR spectra of the biochar materials (Figure 2) showed that the four types of sludge biochar had similar surface functional groups. A characteristic peak at 3429 cm^−1^ was observed, primarily due to the stretching vibration of hydroxyl groups (-OH) from water molecules on the biochar surface [24]. A characteristic peak at 1637 cm^−1^, corresponding to the stretching vibrations of aryl-C=C bonds, has been observed. This indicates the presence of aromatic condensation occurring within the biochar generated from organic matter [25]. A peak at 1051 cm^−1^ was attributed to the vibration of oxygen-containing functional groups (C-O), while a peak at 780 cm^−1^ represented the bending vibration of aromatic C-H bonds [26].

##### X-Ray Diffraction Spectroscopy

**Figure 3:**
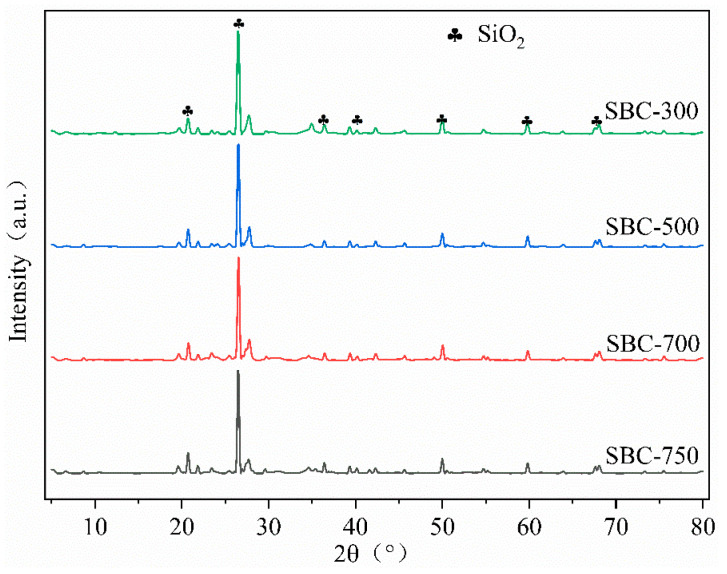
XRD spectra of sludge biochar.

Figure 3 shows the X-ray diffraction (XRD) spectra of the sludge biochar. The results indicate that the crystalline phases of the biochar samples prepared at different pyrolysis temperatures were similar. As the pyrolysis temperature increased, the crystalline phases of the samples did not change significantly, with the main component being silicon dioxide (SiO_2_) (PDF Card: 97-003-9830), consistent with the Si element observed in the EDS images.

Combining the above results, the sludge biochar prepared at 700 °C has the largest specific surface area and exhibits the best adsorption effect on fluorides. Therefore, SBC-700 was selected for lanthanum modification to further investigate its adsorption performance for fluorides.

### 3.2. Optimization of Preparation Conditions and Characterization of Lanthanum-Loaded Biochar

#### 3.2.1. Preparation of Lanthanum-Loaded Biochar

Orthogonal experiments were conducted to explore the preparation of lanthanum-loaded sludge biochar and its adsorption capacity for fluorides under different factor level conditions, as shown in Table 2. According to the range analysis results of the adsorption capacity (R value), the order of factors affecting the adsorption capacity of biochar is B > D > C > A. The optimal experimental conditions are B_2_D_3_C_2_A_3_, which corresponds to a solution pH of 9, a lanthanum nitrate concentration of 0.08 mol/L, a solid-to-liquid ratio of 1:20 (g:mL), and a modification temperature of 55 °C. After supplementing these preparation conditions, the adsorption capacity of the biochar was measured to be 7.582 mg/g, which is about 10 times higher than the adsorption capacity of the original biochar. This lanthanum-loaded biochar is named La-SBC-700.

#### 3.2.2. Characterization of Lanthanum-Loaded Biochar

##### Comparison of SEM-EDS Images

**Figure 4:**
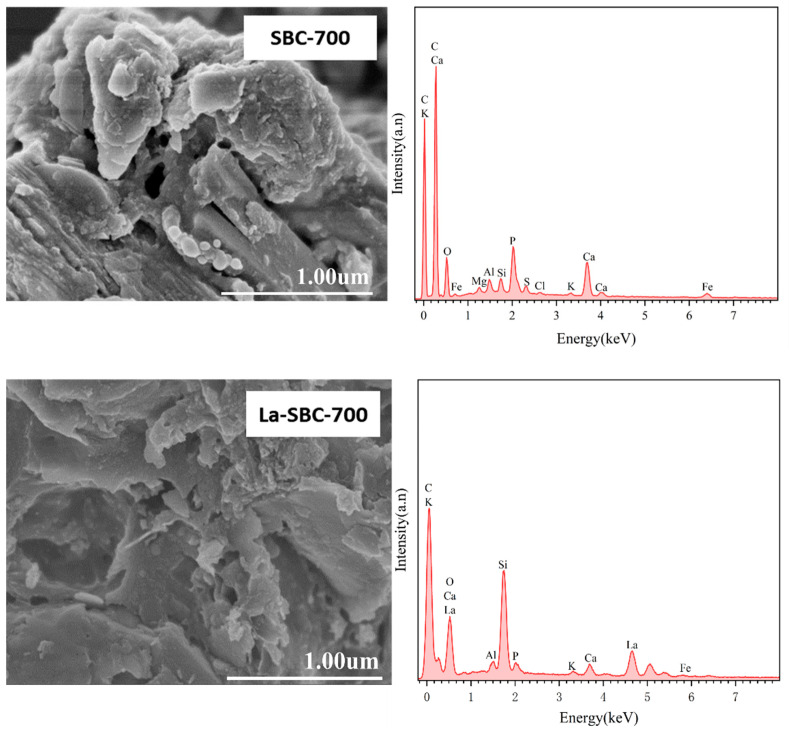

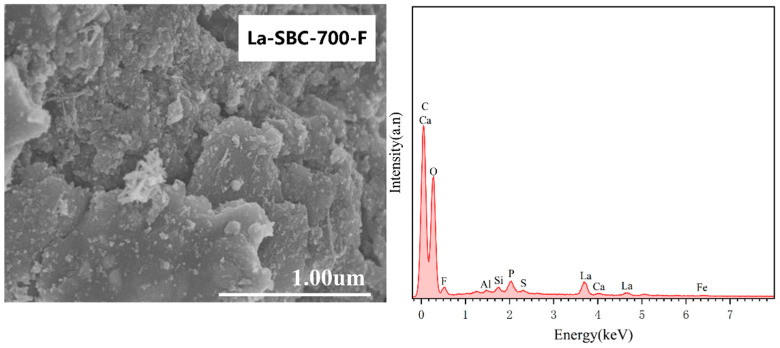
SEM-EDS spectra of La-SBC-700 before and after modification and adsorption.

SEM-EDS analysis was conducted on the lanthanum-loaded biochar (La-SBC-700) to compare the changes in surface structure and elemental composition before and after modification (Figure 4). The SEM images of La-SBC-700 revealed the presence of small particles and columnar structures on the material’s surface. The EDS spectra showed the appearance of La, indicating that lanthanum was successfully loaded onto the biochar. Simultaneously, the surface of the lanthanum-loaded biochar was observed to develop prominent flocculent deposits. This observation, coupled with the detection of the F element in the EDS spectra, suggests that fluoride ions have been effectively adsorbed by the lanthanum-loaded biochar.

##### Specific Surface Area and Pore Size Analysis

The specific surface area, pore volume, and other parameters of La-SBC-700 before and after adsorption were measured and compared with the structural parameters of SBC-700, as shown in Table 3. The results indicate that the external surface area, average pore diameter, and total pore volume of La-SBC-700 all increased, while the micropore volume significantly decreased. This is because there are numerous ionic adsorption sites on the surface and within the pores of the biochar. After combining with the lanthanum solution, both the surface and internal pores are loaded with lanthanum-containing materials, which are primarily present in the micropores. This leads to an increase in the average pore diameter and a decrease in the micropore volume of the lanthanum-loaded biochar [27].

However, the adsorption performance of La-SBC-700 for fluorides is superior to that of the pristine biochar. This may be due to the porous structural characteristics of the biochar, which facilitate the loading of lanthanum. Lanthanum has a strong affinity for fluoride, and under the combined effects of multiple factors, the adsorption rate of fluorides by the lanthanum-loaded biochar is enhanced. After the lanthanum-loaded biochar adsorbed fluorides (La-SBC-700-F), the specific surface area, external surface area, average pore diameter, and total pore volume increased, while the micropore area and micropore volume further decreased. This may be due to the combination of loaded lanthanum-based compounds with fluoride, resulting in the appearance of a large amount of flocculent material in the SEM images.

##### Infrared Spectral Analysis

**Figure 5:**
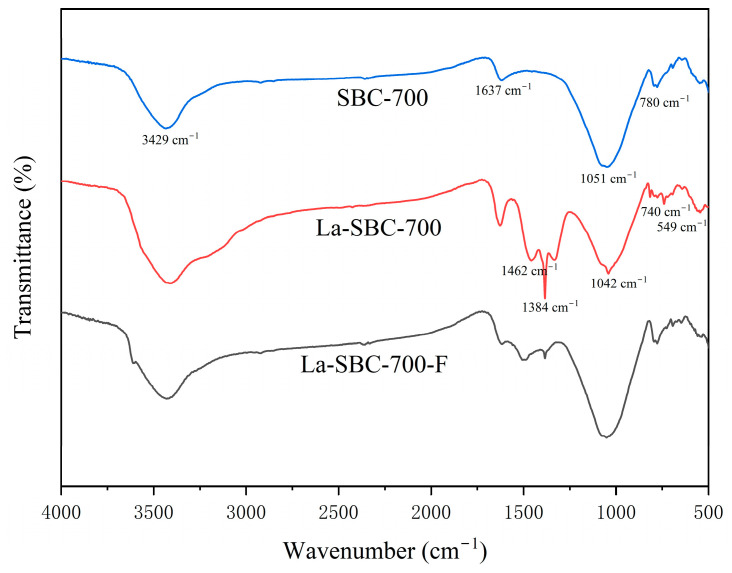
FTIR image of SBC-700, La-SBC-700, and La-SBC-700-F.

Figure 5 indicates that La-SBC-700 exhibits new vibrational characteristic peaks at 549 cm^−1^, 740 cm^−1^, 1042 cm^−1^, and 1462 cm^−1^. The absorption peaks at 549 cm^−1^ and 740 cm^−1^ are attributed to the stretching vibrations of La-O bonds [28]. The characteristic absorption peaks observed at 1042 and 1462 cm^−1^ can be attributed to the formation of carbonate groups resulting from the reaction of La(OH)_3_ with CO_2_ in the air [29]. At 1384 cm^−1^, a characteristic peak for the -NO_2_ groups is exhibited, which is inferred to be introduced due to the use of lanthanum nitrate as a modifying agent [30].

The above results all indicate that lanthanum loading has been successfully carried out on the SBC-700 material. Furthermore, after the lanthanum-loaded biochar adsorbed fluorides (La-SBC-700-F), the absorption peaks at 549 cm^−1^ and 740 cm^−1^ weakened or disappeared, indicating that the La-O bonds in the material participated in the adsorption process of the fluorides. However, the vibration of the La-F bonds formed in LaF_3_ could not be observed in the mid-infrared region [31]. At the same time, it is speculated that ion exchange is an important adsorption mechanism for the adsorption of fluoride by lanthanum-loaded biochar [32].

##### Comparison of XRD Patterns

**Figure 6:**
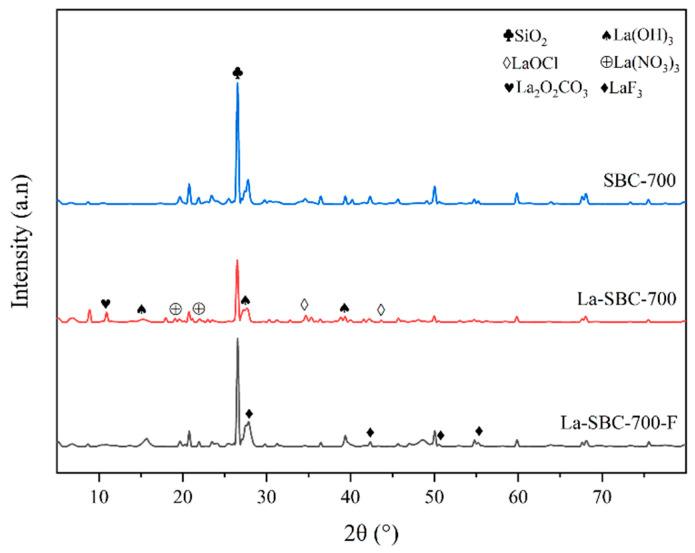
XRD image of SBC-700 and La-SBC-700.

From Figure 6, it is evident that the XRD spectrum of lanthanum-loaded biochar La-SBC-700 exhibits characteristic diffraction peaks of lanthanum-containing oxides such as La_2_O_2_CO_3_ (PDF Card: 00-037-0804), La(OH)_3_ (PDF Card: 97-016-7480), La(NO_3_)_3_ (PDF Card: 00-024-11120), and LaOCl (PDF Card: 97-004-0297). These lanthanum-containing substances all have a certain affinity for fluorides, which contributes to the enhancement of their removal efficiency [33]. All these results indicate that lanthanum-containing compounds have been loaded onto the surface of SBC-700. After La-SBC-700 adsorbed fluorides, some characteristic diffraction peaks of the lanthanum-based compounds weakened or disappeared, and characteristic peaks of LaF_3_ appeared near 28°, 42°, 51°, and 55°, further confirming that the lanthanum-loaded biochar successfully adsorbed the fluorides.

### 3.3. Batch Adsorption Experiments

#### 3.3.1. Effects of Initial Solution pH on Adsorption Performance

**Figure 7:**
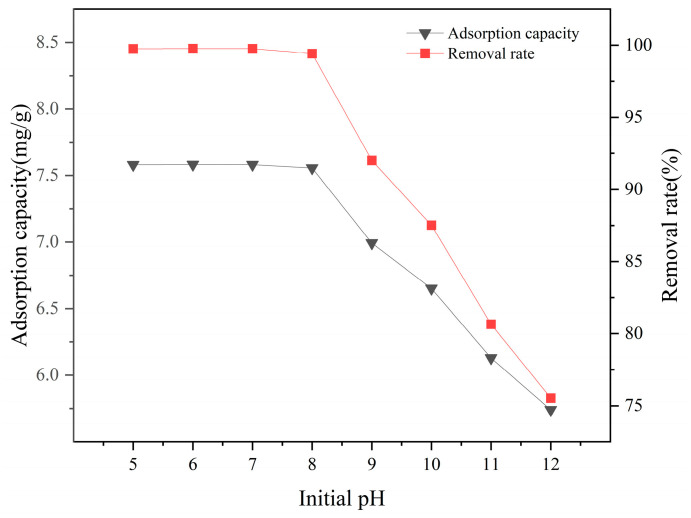
Effect of initial solution pH on the removal rate and adsorption capacity of adsorbed fluoride by La-SBC-700.

The influence of solution pH on the adsorption rate of La-SBC-700 is shown in Figure 7. The removal rate of fluorides by La-SBC-700 is stable when the pH value is between 5 and 8, with the highest removal rate occurring at pH 7. Within this pH range, the leaching of La^3+^ from the biochar and its combination with F^−^ can be neglected, making it suitable for the pH levels of most geothermal waters [11]. However, within the pH range of 9 to 12, the removal rate of fluorides continuously decreases. This is attributed to the fact that the pH value of the aqueous solution is much higher than the equipotential point of La-SBC-700. As a result, the hydroxyl groups on the surface of the biochar become deprotonated and negatively charged, creating electrostatic repulsion with the negatively charged fluorides. In addition, the high concentration of OH^−^ in the solution competes with fluorides for the active adsorption sites on the material [34], inhibiting the adsorption of fluorides [35]. Therefore, subsequent experiments were all conducted at pH = 7.

#### 3.3.2. Effects of F^−^ Concentration on Adsorption Performance

**Figure 8:**
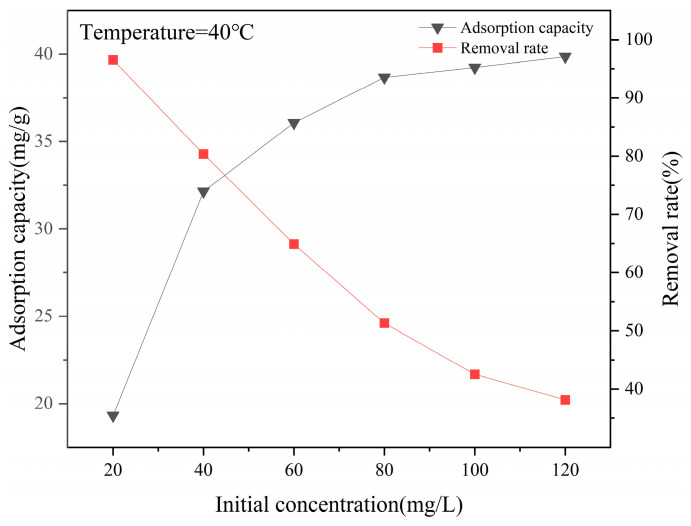
Effect of initial solution concentration on the removal rate and adsorption capacity of adsorbed fluoride by La-SBC-700.

As shown in Figure 8, the adsorption capacity of La-SBC-700 for fluorides increases with the initial fluoride concentration and then tends to level off. With the increase in the fluoride concentration, the adsorption capacity of La-SBC-700 increases from 19.375 mg/g to 40.369 mg/g. This is because when the initial fluoride concentration in the solution is low, there are ample active adsorption sites on the surface of the biochar material to fully adsorb the fluorides. However, as the fluoride concentration increases, the number of fluoride ions that can be combined with the active adsorption sites on the biochar surface is limited, and the adsorption capacity for fluorides also tends to stabilize.

#### 3.3.3. Effects of La-SBC-700 Dosage on Adsorption Performance

**Figure 9:**
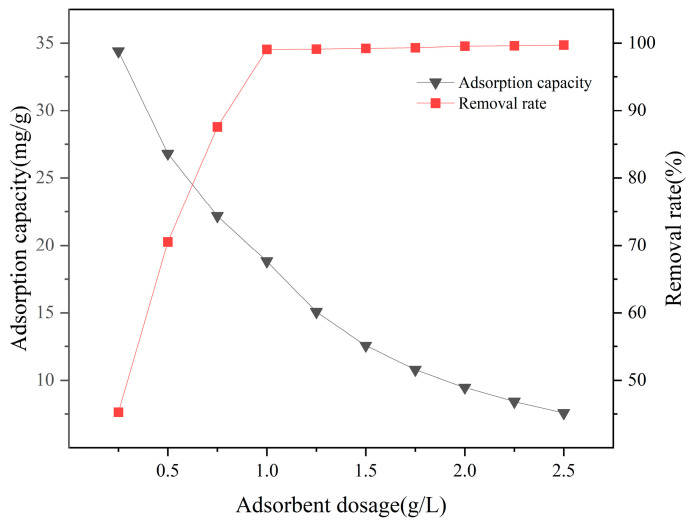
Effect of adsorbent dosage on the removal rate and adsorption capacity of adsorbed fluoride by La-SBC-700.

Figure 9 indicates that at a certain fluoride concentration (19.0 mg/L), as the dosage of La-SBC-700 increases from 0.25 g/L to 2.5 g/L, the removal rate of fluorides first rises linearly and then approaches equilibrium, increasing from an initial 45.26% to 99.08%. The adsorption reaches equilibrium when the dosage is at 1 g/L. However, with the increase in the dosage of La-SBC-700, the adsorption capacity decreased from 34.398 mg/g to 7.579 mg/g. This is due to the constant amount of fluoride in the solution. As the amount of adsorbent added increases, the number of idle adsorption active sites also increases, resulting in a decrease in the adsorption amount [36].

#### 3.3.4. Effects of Coexisting Ions on Adsorption Performance

**Figure 10:**
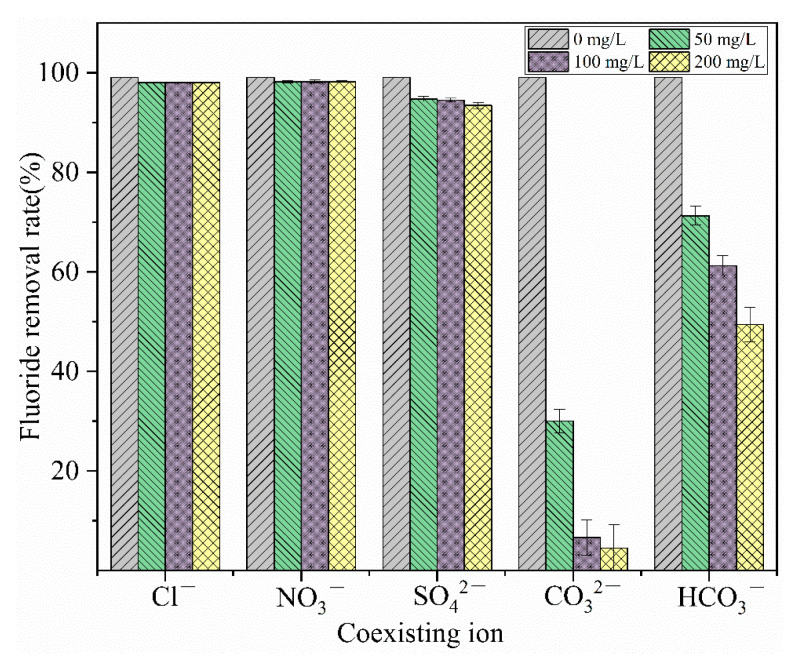
Effect of coexisting ion on the adsorption of fluoride.

Figure 10 shows that ${NO}_{3}^{-}$ and Cl^−^ have almost no effect on the removal of fluorides, but ${CO}_{3}^{2-}$, ${SO}_{4}^{2-}$, and ${HCO}_{3}^{-}$ have varying degrees of influence on the removal of fluorides, with the extent of impact increasing as the concentration of these ions increases. The ${SO}_{4}^{2-}$ ion, due to its high charge density, leads to a suppression of the adsorption rate of fluorides. In addition to charge density, the reaction product La_2_(CO_3_)_3_ has a lower solubility than LaF_3_. Moreover, ${CO}_{3}^{2-}$ undergoes a secondary hydrolysis, generating a large amount of OH^−^, which raises the pH of the solution to an alkaline level [37], causing a sharp decrease in the removal rate of fluorides [38]. When the concentration of ${CO}_{3}^{2-}$ increases to 200 mg/L, the removal rate of fluorides is only 4.47%. The interference from ${HCO}_{3}^{-}$ stems from the hydrolysis reaction that also produces ${CO}_{3}^{2-}$, which competes with fluorides for adsorption.

#### 3.3.5. Adsorption Kinetics

**Figure 11:**
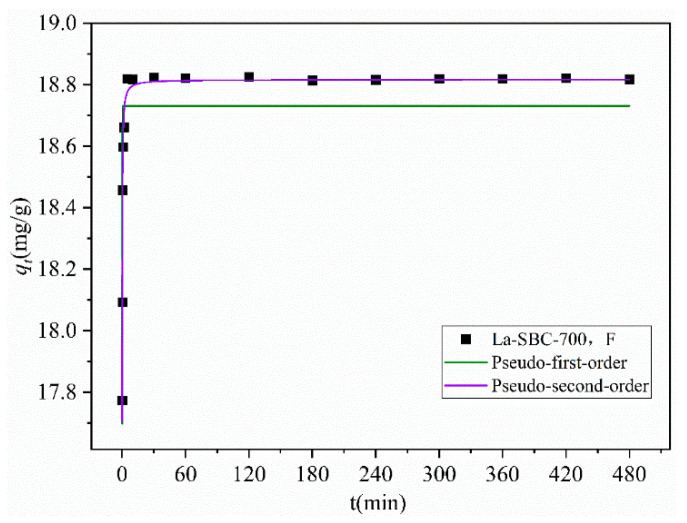
Kinetic fitting curves.

As shown in Table 4 and Figure 11, the adsorption amount of fluorides by the adsorbent rapidly increased within the first 5 min of the reaction, reaching adsorption equilibrium at 60 min with a saturated adsorption capacity of 18.818 mg/g. This value is close to the calculated equilibrium adsorption capacity *q_e,cal_* of 18.817 mg/g obtained through fitting. Furthermore, the fitting correlation coefficient (R^2^ = 0.9975) for the Quasi-secondary-order kinetic model is higher than that (R^2^ = 0.6159) for the Quasi-primary-order kinetic model, indicating that the adsorption process of fluorides onto La-SBC-700 is primarily governed by chemical adsorption, which is the main controlling factor for the adsorption rate [38].

#### 3.3.6. Adsorption Isotherms

**Figure 12:**
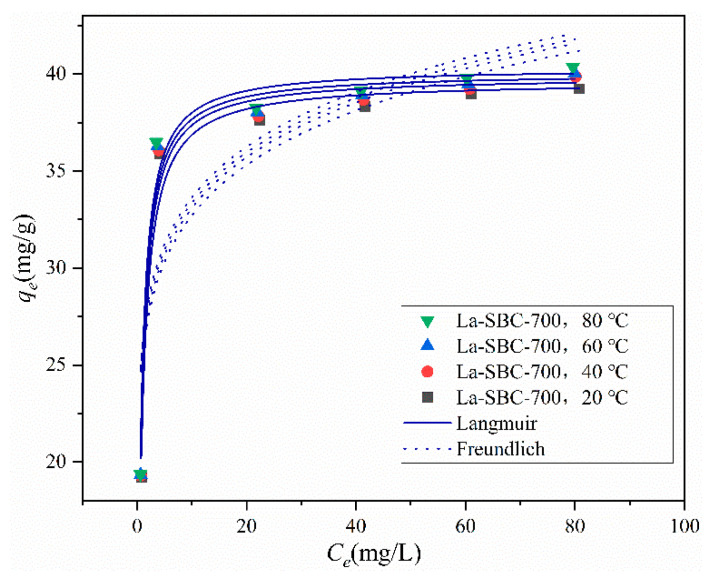
Adsorption isotherms.

As shown in Table 5 and Figure 12, the fitting coefficient R^2^ for the Langmuir model ranges from 0.9676 to 0.9754, which is higher than that for the Freundlich model. Moreover, the maximum adsorption capacity simulated by the Langmuir model is 40.338 mg/g. This indicates that the adsorption behavior of fluorides onto La-SBC-700 is better described by the Langmuir model, suggesting that the process is primarily dominated by monolayer adsorption [39]. The simulated maximum adsorption capacity does not vary significantly across different temperatures, ranging from 39.634 to 40.338 mg/g, which is more than double the adsorption capacity of lanthanum-modified pomelo peel biochar, at 19.86 mg/g [13], indicating an enhanced adsorption performance by a factor of two.

Previous studies have found that the adsorption capacity of traditional metal or multi-metal composite adsorbents for fluoride ranges from 16.30 to 31.72 mg/g [40,41,42]. The adsorption capacity of biochar derived from common solid waste ranges from 18.52 to 30.49 mg/g [15,19,43]. In this study, La-SBC-700 exhibited a maximum adsorption capacity of 40.338 mg/g, which, although not as high as that of La/Fe/Al-RSBC [16], is still significant. Its simple preparation process and low cost make it suitable for the low-carbon fixation of municipal sludge and hold great potential for the removal of fluorides from water. To have a more intuitive understanding of the adsorption capacity of different materials for fluorine, we have cited relevant literature, as shown in Table 6.

#### 3.3.7. Adsorption Thermodynamics

Using the data from the isothermal adsorption experiments, the equilibrium constant *K_0_* and the standard Gibbs free energy change Δ*G*^0^ at different temperatures were calculated using Equations (7) and (8), respectively. Subsequently, by plotting *lnK_b_* against *1*/*T* according to Equation (9), the values of Δ*H*^0^ (standard enthalpy change) and Δ*S*^0^ (standard entropy change) were determined from the slope and intercept of the plot. From Table 7, it is evident that Δ*H*^0^ > 0, indicating that the adsorption process of fluorides by La-SBC-700 is endothermic. The adsorption process has Δ*G*^0^ < 0, suggesting that the adsorption behavior is spontaneous, and the value of Δ*G*^0^ decreases with increasing temperature, indicating that an increase in temperature favors the spontaneous reaction. Δ*S*^0^ > 0, indicating that the degree of disorder at the solid/liquid interface of the adsorbent increases after adsorption occurs, a conclusion that is consistent with the findings of He [44] and Raghav [45] et al.

#### 3.3.8. Adsorption Experiments of Fluoride in Real Geothermal Water

To explore the practical application potential of La-SBC-700, adsorption experiments were conducted using actual water samples labeled S1, S2, G1, G2, and G3, which were collected from Yangbajing Town, Damxung County, Lhasa City, Tibet, China. The results when the dosage of La-SBC-700 was 2 g/L are shown in Table 8. The removal rate of fluorides from the actual geothermal water samples by La-SBC-700 was higher than 98%. However, the dosage of La-SBC-700 was increased compared to the simulated experiments. This is because the pH value of the water samples was relatively high; the high concentration of OH^−^ competed with fluorides for the adsorption sites on the material. Additionally, the presence of certain amounts of ions such as ${SO}_{4}^{2-}$ and ${HCO}_{3}^{-}$ in geothermal water generally inhibits the removal of fluorides.

### 3.4. Adsorption Mechanism

Based on the above kinetic models, isothermal models, and characterization analysis results of the fluoride adsorption process, Figure 11 shows that the adsorption process is composed of two stages: rapid adsorption and adsorption equilibrium. In the first stage, the biochar surface has ample pore channels and abundant functional groups, allowing fluoride to quickly occupy the material’s pores and adsorption sites. As adsorption proceeds, the number of pore channels and functional groups in the carbon material gradually decreases, entering the adsorption equilibrium stage. Table 4 shows that the Quasi-second-order kinetic model fits the adsorption process more closely, and the isothermal model also conforms better to the Langmuir equation. In summary, the adsorption process of fluoride on lanthanum-loaded biochar is likely primarily chemisorption. The reactions on the surface of La-SBC-700 material control the chemisorption, and it is speculated that there is electron exchange between the fluoride and the adsorbent material.

The infrared spectrum of La-SBC-700-F (Figure 5) shows a significant shift in the -OH characteristic peak at 3429 cm^−1^ compared to that before adsorption, likely resulting from ion exchange interactions between -OH and F^−^ ions [46]. Concurrently, the La-O group signatures at 740 cm^−1^ and 549 cm^−1^ show significant attenuation or complete disappearance. Although LaF_3_ was not detected in spectral analyses, the presence of distinct LaF_3_ diffraction peaks in the XRD pattern of La-SBC-700-F confirms that precipitation is also one of its adsorption mechanisms.

It is worth noting that after La-SBC-700 adsorbs F^−^, the material surface is extensively covered with massive, spherical substances. The specific surface area increases significantly, while the micropore area decreases markedly. This may be due to the entry of F^−^ into the pore structure of La-SBC-700 and its subsequent combination with lanthanum-based compounds to form lanthanum fluoride compounds, which is consistent with the characteristics of pore filling [47].

The comprehensive analysis demonstrates that fluoride adsorption by La-SBC-700 predominantly involves four synergistic mechanisms: (1) electrostatic attraction, (2) ion exchange, (3) chemical precipitation, and (4) pore filling effects.

## 4. Conclusions

(1) Utilizing sludge as the raw material, orthogonal experiments were employed to determine the optimal preparation conditions for La-SBC-700, which are a lanthanum nitrate concentration of 0.08 mol/L, a pH of 9 for the lanthanum nitrate solution, a solid-to-liquid ratio of SBC-700 to lanthanum nitrate solution of 1:20 (g:mL), and a modification temperature of 55 °C. These conditions result in an adsorption capacity approximately ten times higher than that of the original biochar (SBC).
(2) The Quasi-secondary-order kinetics and the Langmuir isothermal adsorption model are more consistent with the adsorption process of F^−^ onto La-SBC-700. Within the temperature range of 20 to 80 °C, the maximum adsorption capacity of La-SBC-700 ranges from 39.634 to 40.338 mg/g, indicating that the lanthanum-loaded biochar has good adsorption performance for fluorides. Thermodynamic studies indicate that the adsorption process of fluoride onto La-SBC-700 is a spontaneous endothermic reaction. Moreover, as the adsorption reaction proceeds, the degree of disorder at the solid/liquid interface of the adsorbent increases, affecting the surface structure of the adsorbent.
(3) At a dosage of 2 g/L of La-SBC-700, this biochar material achieved a fluoride removal rate higher than 98% in geothermal water from various sampling sites, demonstrating excellent defluoridation performance.

## Figures and Tables

**Table 1:** Specific surface area, average pore size, and pore volume of sludge biochar.

Biochar Type	Specific Surface Area (m^2^/g)	Micropore Area (m^2^/g)	External Surface Area (m^2^/g)	Average Pore Diameter (nm)	Total Pore Volume (cm^3^/g)	Micropore Volume (cm^3^/g)
SBC-300	1.184	0.250	0.934	18.493	0.005	0.00009
SBC-500	7.384	7.350	0.034	4.851	0.009	0.00414
SBC-700	39.632	36.143	3.489	3.158	0.031	0.01869
SBC-750	10.254	9.428	0.826	6.028	0.013	0.00484

**Table 2:** Results of orthogonal experiments on lanthanum-loaded sludge biochar.

Serial No.	Temperature (°C)	Solution pH	Solid–Liquid Ratio (g:mL)	Lanthanum Nitrate Concentration (mol/L)	Fluoride Adsorption (mg/g)
	A	B	C	D	
1	35	7	1:15	0.04	6.10
2	35	9	1:20	0.06	7.56
3	35	11	1:25	0.08	6.27
4	45	7	1:20	0.08	6.58
5	45	9	1:25	0.04	6.65
6	45	11	1:15	0.06	5.96
7	55	7	1:25	0.06	6.26
8	55	9	1:15	0.08	7.52
9	55	11	1:20	0.04	6.20
k_1_	6.643	6.313	6.527	6.320	
k_2_	6.400	7.247	6.780	6.593	
k_3_	6.660	6.143	6.397	6.790	
R	0.260	1.104	0.383	0.470	

**Table 3:** Specific surface area, average pore size, and pore volume of biochar and lanthanum-loaded biochar.

Biochar Name	Specific Surface Area (m^2^/g)	Micropore Area (m^2^/g)	External Surface Area (m^2^/g)	Average Pore Diameter (nm)	Total Pore Volume (cm^3^/g)	Micropore Volume (cm^3^/g)
SBC-700	39.632	36.143	3.489	3.158	0.031	0.01869
La-SBC-700	14.21	5.198	9.012	21.737	0.077	0.00213
La-SBC-700-F	20.462	1.189	19.273	22.586	0.116	0.00042

**Table 4:** Quasi-primary and quasi-secondary kinetic parameters of La-SBC-700 for fluoride adsorption.

Material	*q_e,exp_* /(mg/g)	Quasi-Primary-Order Kinetics			Quasi-Secondary-Order Kinetics		
		k_1_ /(min^−1^)	*q_e,cal_* /(mg/g)	R^2^	k_2_ /(g·(mg·min)^−1^)	*q_e,cal_* /(mg/g)	R^2^
La-SBC-700	18.818	17.039	18.731	0.6159	4.977	18.817	0.9775

**Table 5:** Adsorption isothermal data for fluoride adsorption on La-SBC-700.

Material	Temperature (°C)	Langmuir			Freundlich		
		*q_m_* (mg/g)	*K_L_* (L/mg)	R^2^	*K_F_* (mg/g)	n	R^2^
La-SBC-700	20	39.635	1.327	0.9676	25.354	9.046	0.6788
	40	39.881	1.481	0.9754	25.682	9.059	0.6998
	60	40.071	1.567	0.9749	25.975	9.211	0.6957
	80	40.369	1.626	0.9727	26.233	9.262	0.6932

**Table 6:** Adsorption capacity of different materials for fluorine.

Material	*q_m_* (mg/g)	References
Aluminum Oxide Material	16.300	[41]
Y-Zr-Al Composite Material	31.000	[42]
Mg-Al-La Metal Oxide	31.720	[43]
Al-Fe Loaded Tea Residue Biochar	18.520	[44]
La Modified Pomelo Peel Biochar	19.860	[15]
ALCS-Fe-Al Composite Material	30.490	[19]
La-SBC-700 Biochar	40.338	this article

**Table 7:** Thermodynamic parameters related to fluoride adsorption on La-SBC-700.

Item	Temperature (K)	K_0_	Δ*G*^0^ (kJ/mol)	Δ*H*^0^ (kJ/mol)	Δ*S*^0^ kJ/(mol·k)
La-SBC-700	293.15	24.2302	−7.769	3.483	0.0386
	313.15	27.8892	−8.665		
	333.15	29.6701	−9.39		
	353.15	30.9795	−10.081		

**Table 8:** Removal experiments of fluoride in real geothermal water.

Water Type	Code	Fluoride Concentration (mg/L)	pH	Removal Rate
Hot Spring Water	S1	5.44	7.48	99.41%
Hot Spring Water	S2	8.76	8.69	99.06%
Geothermal Water Outlet	G1	11.49	9.17	98.11%
Geothermal Water Outlet	G2	10.32	9.08	99.12%
Geothermal Water Outlet	G3	11.45	8.76	98.96%

## Data Availability

The original contributions presented in this study are included in the article. Further inquiries can be directed to the corresponding author.
